# Before the Polymerase Chain Reaction Test: Where Surveillance Truly Begins

**DOI:** 10.4269/ajtmh.26-0112

**Published:** 2026-04-23

**Authors:** Carlos Bastidas-Caldes

**Affiliations:** ^1^ School of Medicine, Universidad Espíritu Santo, Samborondón, Ecuador;; ^2^ Instituto Nacional de Biodiversidad, Quito, Ecuador

By the fourth day in the humid forests of coastal Ecuador, I could no longer see my knuckles. My hands were swollen from mosquito bites, and my feet were covered in blood-filled blisters. I had not slept more than 4 hours per night since we arrived. The field biologists I was working with, however, continued as if this were expected. We had traveled along Ecuador’s tropical coast to remote forested areas of the Esmeraldas and Manabí Provinces to sample wild mammals. The work formed part of a study on *Histoplasma capsulatum*.

I had led laboratory-based research before, but this was my first sustained immersion in tropical field biology. During those days in the forest, I began to understand something that had always seemed abstract to me in the laboratory: primary pandemic prevention does not start in a laboratory; it begins with ecological understanding, interdisciplinary collaboration, and sustained investment in field surveillance.

We stayed in abandoned wooden cabins surrounded by dense vegetation. The floors were layered with bat guano. There was no potable water, no filtration system, and no reliable electricity. We cooked every meal ourselves over improvised stoves, using limited supplies and visibly contaminated water. Only later did I learn that during longer expeditions, field teams often hire someone to cook, not for comfort, but because the combination of setting nets, checking traps, processing specimens, preserving tissues, documenting data, and preparing meals is physically unsustainable. Field science requires division of labor. Before this experience, I had underestimated that reality.

Fieldwork followed a strict rhythm. In the mornings and afternoons, we walked through primary and secondary forests to establish transects. Before sunset, we installed mist nets for bats and Sherman traps for small mammals. At dusk, activity intensified.

From 6 pm until nearly 2 am, we checked nets and traps repeatedly. Headlamps were essential, but they created dense halos of insects that made breathing difficult. Mosquitoes entered our eyes and nostrils. I covered my face with a cloth in an attempt to create a barrier. Turning off the light was not possible; the forest floor was uneven, thick with vegetation, and potentially hazardous.

Captured animals were processed in accordance with strict ethical and museum protocols. Fecal samples, oral swabs, liver tissue, and intestinal segments were each collected systematically. Some animals met the established criteria for scientific collections and were euthanized so that their skins and skeletal material could later be used for taxonomic identification. Entire specimens were preserved in ethanol. The work demanded precision and respect. Sleep began around 3 am. By 6:30 am, we were awake again.

By the fourth day, the fatigue became limiting. The swelling in my hands made delicate movements painful. The blisters on my feet throbbed inside my boots, some broken open and stained with blood. Night walks were the most difficult. The forest floor was uneven and hidden beneath dense vegetation, and exhaustion made every step uncertain. I had always feared snakes. As a microbiologist with public health training, I had seen the consequences of envenomation, and that knowledge remained present with each step in the darkness. I remained in camp for one day to process samples while the field biologists continued in the forest. They did not consider the workload extraordinary. For them, it was simply the cost of performing science properly.

Months later, laboratory analyses would confirm the presence of fungal DNA in several of the collected specimens. However, the most important realization for me did not occur later in the laboratory, in front of the polymerase chain reaction (PCR) machine; it occurred there in the forest. After days of exhaustion, walking through dense vegetation, carrying equipment, and working under difficult conditions, I began to understand that the samples that eventually arrive in the laboratory are the final step in a long, physically demanding process conducted by field scientists working far from infrastructure and institutional visibility.

Surveillance of tropical diseases does not begin with sequencing data or statistical models. It begins in forests and agricultural interfaces, where wildlife, domestic animals, and humans intersect. It depends on ecologists, taxonomists, and field technicians who endure physical strain to obtain representative samples.

In journals and conferences, we discuss surveillance frameworks and One Health strategies in conceptual terms. In the forest, those same frameworks acquire meaning; they have blisters, fatigue, mosquito bites, and long nights under headlamps.

This work cannot be accomplished in isolation. Physicians and laboratory scientists rely on the commitment and expertise of field biologists, just as ecological surveillance depends on clinical and microbiological interpretation. Each percentage reported in a scientific publication reflects nights without sleep, repeated transects, careful specimen handling, and coordinated teamwork.

I returned from the forest physically exhausted but intellectually transformed. Wildlife surveillance is not an auxiliary component of infectious disease research; it is foundational. The prevention of future pandemics may depend as much on strengthening field capacity and interdisciplinary respect as on technological innovation. Before the PCR test, there is the forest and the people who make surveillance possible.

